# Usability of Electronic Patient-Reported Outcome Measures for Older Patients With Cancer: Secondary Analysis of Data from an Observational Single Center Study

**DOI:** 10.2196/49476

**Published:** 2023-09-21

**Authors:** David Riedl, Jens Lehmann, Maria Rothmund, Daniel Dejaco, Vincent Grote, Michael J Fischer, Gerhard Rumpold, Bernhard Holzner, Thomas Licht

**Affiliations:** 1 Ludwig Boltzmann Institute for Rehabilitation Research Vienna Austria; 2 University Hospital of Psychiatry II Department of Psychiatry, Psychotherapy Psychosomatics and Medical Psychology Medical University of Innsbruck Innsbruck Austria; 3 Institute of Psychology University of Innsbruck Innsbruck Austria; 4 Department for Otorhinolaryngology, Head and Neck Surgery Medical University of Innsbruck Innsbruck Austria; 5 Vamed Rehabilitation Center Kitzbühel Kitzbühel Austria; 6 Evaluation Software Development (ESD) Innsbruck Austria; 7 University Hospital of Psychiatry I Department of Psychiatry, Psychotherapy Psychosomatics and Medical Psychology Medical University of Innsbruck Innsbruck Austria; 8 Oncological Rehabilitation Center Sankt Veit im Pongau Austria; 9 Paracelsus Medical University Salzburg Austria

**Keywords:** patient-reported outcomes, completion rate, geriatric, age, patient reported, elderly, older adults, older adult, cancer, oncology, survivor, survivors, questionnaire, questionnaires, self-reported, geriatrics, gerontology, survey, surveys, mobile phone

## Abstract

**Background:**

Patient-reported outcomes are considered the gold standard for assessing subjective health status in oncology patients. Electronic assessment of patient-reported outcomes (ePRO) has become increasingly popular in recent years in both clinical trials and practice. However, there is limited evidence on how well older patients with cancer can complete ePRO assessments.

**Objective:**

We aimed to investigate how well adult patients with cancer of different age ranges could complete ePRO assessments at home and in a treatment facility and to identify factors associated with the ability to complete questionnaires electronically.

**Methods:**

This retrospective longitudinal single-center study involved survivors of cancer who participated in inpatient rehabilitation. Patients completed ePRO assessments before rehabilitation at home (T1) and after rehabilitation at the facility (T2). We analyzed the rate of patients who could complete the ePRO assessment at T1 and T2, the proportion of patients who required assistance, and the time it took patients to complete standardized questionnaires. Multivariate logistic regression analyses were conducted to identify predictors of ePRO completion rate and the need for assistance.

**Results:**

Between 2017 and 2022, a total of 5571 patients were included in this study. Patients had a mean age of 60.3 (SD 12.2) years (range 18 to 93 years), and 1135 (20.3%) of them were classified as geriatric patients (>70 years). While more than 90% (5060/5571) of all patients completed the ePRO assessment, fewer patients in the age group of >70 years (924/1135, 81.4% at T1 vs 963/1135, 84.8% at T2) completed the assessment. Approximately 19% (1056/5571) of patients reported a need for assistance with the ePRO assessment at home, compared to 6.8% (304/4483) at the institution. Patients older than 70 years had a significantly higher need for assistance than those in younger age groups. Moreover, a gender difference was observed, with older women reporting a higher need for assistance than men (71-80 years: women requiring assistance 215/482, 44.6% vs men 96/350, 27.4%; *P*<.001 and >80 years: women 102/141, 72.3% vs men 57/112, 50.9%; *P*<.001). On average, patients needed 4.9 (SD 3.20) minutes to remotely complete a 30-item questionnaire (European Organization for the Research and Treatment of Cancer Quality of Life Questionnaire) and patients in the older age groups took significantly longer compared to younger age groups. Lower age and higher physical functioning were the clearest predictors for both the ePRO completion rate and the need for assistance in the multivariate regression analysis.

**Conclusions:**

This study’s results indicate that ePRO assessment is feasible in older individuals with cancer, but older patients may require assistance (eg, from relatives) to complete home-based assessments. It may be more feasible to conduct assessments in-house in this population. Additionally, it is crucial to carefully consider which resources are necessary and available to support patients in using ePRO devices.

## Introduction

In the last decades, advances in cancer diagnostics and treatment have resulted in improved life expectancies [1-3], leading to an increasing number of survivors of cancer of higher age [1,4], who are at significant risk for disease- and treatment-related late effects and decreased health-related quality of life (HRQOL) [5-9]. In this context, cancer rehabilitation becomes increasingly important.

Cancer rehabilitation aims to restore psychosocial functioning and reduce symptom burden [6,7] and typically consists of a multimodal and interdisciplinary treatment approach [8]. Several studies have shown the effectiveness of multimodal cancer rehabilitation in increasing the HRQOL of survivors of cancer [10-16].

To evaluate cancer rehabilitation from the patient’s perspective, patient-reported outcomes (PROs) are the gold standard [17]. Apart from treatment evaluation, PROs can also be used to offer a more individualized and tailored rehabilitation treatment [18], to improve communication between patients and therapists, facilitate treatment continuity and patient-centered interventions as well as participatory decision-making [19]. While traditionally PROs were collected with paper-pencil questionnaires, technological advances in the last decades have facilitated the use of electronic PRO assessments (ePROs).

Electronic data assessment and processing has several benefits. In general, it leads to a higher data quality than paper-pencil assessments [20,21] and allows automatic scoring and graphical representation in real time [22,23]. Especially in pediatric and adolescent patients, ePROs are accepted very well and result in very high response rates [24-26]. However, there is a lack of evidence on how well older patients are capable of completing ePRO assessments [27]. Older patients may face challenges when using digital devices for ePRO assessments, as they have not grown up with such devices [28-30]. Additionally, studies showed that among survivors of cancer over half of the individuals have issues with instrumental activities of daily living [31], while even two-thirds of older survivors of cancer report functional limitations [32]. These impairments can include decreased memory and multitasking abilities as well as difficulties with attention focusing and word-finding [33] and may be associated with cancer-related cognitive impairment, which is one of the most frequently reported side effects mainly after chemotherapy [34]. Since more than half of cancer diagnoses occur in individuals 65 years of age or older, cancer-related cognitive impairment and naturally developing cognitive and functional impairments can add up and may impair the ability of older survivors of cancer to partake in relatively complex tasks such as the completion of a web-based questionnaire. However, in prior research, we have also observed variations in cognitive functioning levels among patients with distinct cancer diagnoses [10], which may be associated with specific cancer stages or varying treatment protocols. Thus, more evidence is needed to determine whether electronic assessments are suitable for older patients with differing cancer diagnoses, and at what age electronic assessments become increasingly difficult for patients to complete.

Nevertheless, ePRO assessments are increasingly used in routine clinical assessments before and during cancer treatment [35-38], as well as for cancer aftercare [39] and rehabilitation [18]. This study aims to (1) assess the ability of adult patients of different age groups or cohorts to complete routine ePRO assessments before and after inpatient cancer rehabilitation and how long it took patients to complete the questionnaires, (2) evaluate the proportion of patients requiring support to complete ePRO assessments across age groups, and (3) identify predictors for the ability to complete ePRO assessments and the need for support.

## Methods

### Sample and Procedure

This is a secondary exploratory and extended analysis of data collected for the evaluation of inpatient cancer rehabilitation, which has been reported elsewhere [10]. The data used in this study are part of an ongoing routine clinical data collection at the Oncological Rehabilitation Center Sankt Veit im Pongau, Austria. The sample consisted of adult survivors of cancer who had completed their active oncological treatment prior to admission to inpatient rehabilitation. Data were collected before the start of treatment via a web-based patient portal using the “Computer-based Health Evaluation System (CHES)” [22], where patients completed the questionnaires from home. Before admission to the rehabilitation center (T1), patients received a letter with general information about rehabilitation treatment and authorization to complete an ePRO questionnaire in the patient portal. A cover letter explained that the ePRO data were used to plan patients' treatment intensity (eg, frequency of psycho-oncological treatment) and motivated patients to ask relatives for help if they needed assistance to log into the portal. If patients had not completed the questionnaire by 1 week before admission, they were contacted by a member of the rehabilitation center administration to remind them to complete the ePRO assessment and to offer guidance with technical problems. The second assessment (T2) was conducted at the end of rehabilitation using the same questionnaires as in the first assessment, but this time patients completed the ePRO assessment on tablets provided by the hospital.

### Ethics Approval

After admission to rehabilitation, patients were asked to provide written informed consent for the scientific use of their data. If patients refused, their data were used only for routine care and were not included in this study. This study was reviewed by the Ethics Committee of the Province of Salzburg (415-EP/73/451-2014) and conducted according to the principles of the Declaration of Helsinki.

### Outcome Assessments

The European Organization for the Research and Treatment of Cancer Quality of Life Questionnaire (EORTC QLQ-C30) is a cancer-specific questionnaire, which comprises 30 items. It covers domains of functioning (physical, social, role, emotional, and cognitive), symptom burden (fatigue, nausea or vomiting, pain, dyspnea, sleep disturbances, appetite loss, constipation, diarrhea, and financial impact) as well as a global HRQOL scale. Scales are scored from 0 to 100, with 100 being the best score for functioning scales, whereas 0 identified as the worst score for symptom scales. The Hospital Anxiety and Depression Scale is a 14-item questionnaire to assess psychological distress. It has 2 scales (anxiety and depression), which are summed and range from 0 to 21. Since January 2018, an additional item was added to assess if patients had received help to complete the questionnaire (yes vs no) and if yes, who had assisted them. Finally, we also extracted the time that patients needed to complete the electronic assessment of the EORTC QLQ-C30 from the CHES data logs as an indicator of how easy it was for patients to complete the questionnaires and if they got more confident in completing questionnaires electronically over time (ie, from T1 to T2).

### Statistical Analyses

For the descriptive analysis, patients were divided into 5 groups based on their age: ≤50, 51-60, 61-70, 71-80, and >80 years. Patients aged 71 years or older were categorized as “geriatric” [40]. The relative number of patients who were able to complete the questionnaire from home before admission to the rehabilitation center was defined as the ePRO completion rate at T1 and the relative number of completed ePRO assessments at the end of rehabilitation as the ePRO completion rate at T2. Since data collection before treatment (ePRO assessment via patient portal) and at the end of treatment (ePRO assessment via tablets provided by the rehabilitation center) substantially differed, data for both time points are presented. This could help researchers to determine the mode of assessment or the potential dropout rate for future studies if older patients are being recruited. ePRO completion rates are displayed for the 5 age groups, stratified by sex. Sex differences in each age group were investigated by calculation of chi-squared tests. For 2×2 contingency tables ϕ-values (<0.1=negligible, 0.1-0.3=small, 0.3-0.5= medium, and >0.5=large effects) are reported, while Cramer’s V is given for contingency tables exceeding 2×2 [41,42].

The time in minutes it took patients to complete the EORTC QLQ-C30 was compared between assessments and between groups using *t* tests. We excluded patients who had a completion time of over 60 minutes (which was a strong indication that patients had timed out during the assessments). We analyzed if patients took less time to complete the questionnaire at the second assessment (time T1-time T2) using paired *t* tests. Moreover, we used univariate ANOVA with least significant difference post hoc test (*P* values adjusted using Bonferroni Holms correction) to analyze age group differences in the completion time.

To identify predictors for the ePRO completion rate as well as the need for assistance at T1, logistic regression analyses were calculated. In the first step, the association of the patients’ age (reference category: <50 years), sex, and ICD-10 cancer diagnosis (entered as a categorical variable with breast cancer as reference category) were tested in separate univariate regression analyses. Patients with breast cancer were used as reference category since they are by far the largest patient group and are best represented in all age groups and the treatment of breast cancer is associated with less extensive treatment side effects than other tumor types (eg, head and neck cancer) [10]. Additionally, the association of the EORTC QLQ-C30 subscales with the dependent variables was tested using the backward elimination likelihood ratio method. To facilitate the interpretability of the EORTC QLQ-C30 subscales, scores were transformed to represent changes of 10 points (ie, scores were divided by 10). Thus, odds ratios represent changes of 10 points on each scale, which has been defined as a meaningful difference [43]. Statistically significant associations were then added to the final model, using a logistic regression with the forced entry method. Odds ratios with 95% CI are presented for all predictors. The overall explained variance is described with Nagelkerke *R*². For all calculations, SPSS (version 21.0; IBM Corp) was used.

## Results

### Overview

Between January 2017 and November 2022, a total of 5571 patients were included in this study. Patient mean age was 60.3 (SD 12.2) years, with 1135 (20.3%) classifying as geriatric patients (ie, ≥70 years). Approximately two-thirds of the sample was female and the most frequent cancer diagnoses were breast cancer (2055/5571, 36.9%), hemoblastoses (578/5571, 10.4%), and prostate cancer (471/5571, 8.5%; Table 1).

**Table 1:** Patient characteristics.

		Value, n (%)
**Age (years)**		
	≤50	1025 (18.4)
	51-60	1833 (32.9)
	61-70	1578 (28.3)
	71-80	866 (15.5)
	>80	269 (4.8)
**Sex**		
	Male	1994 (35.8)
	Female	3577 (64.2)
**Cancer entities (International Classification of Diseases, Tenth Revision, codes)**		
	Mamma (C50)	2055 (36.9)
	Hemoblastoses (C81-85, C90-96)	578 (10.4)
	Prostate (C61)	471 (8.5)
	Uterus or ovary (C53-56)	386 (6.9)
	Colon (C18-19)	288 (5.2)
	Head and neck (C00-14; C30-C32)	277 (5)
	Lung (C33-C34)	252 (4.5)
	Rectum (C20-21)	181 (3.2)
	Stomach (C16)	134 (2.4)
	Other	949 (17)

### ePRO Completion Rate Across Age Groups

**Figure 1:**
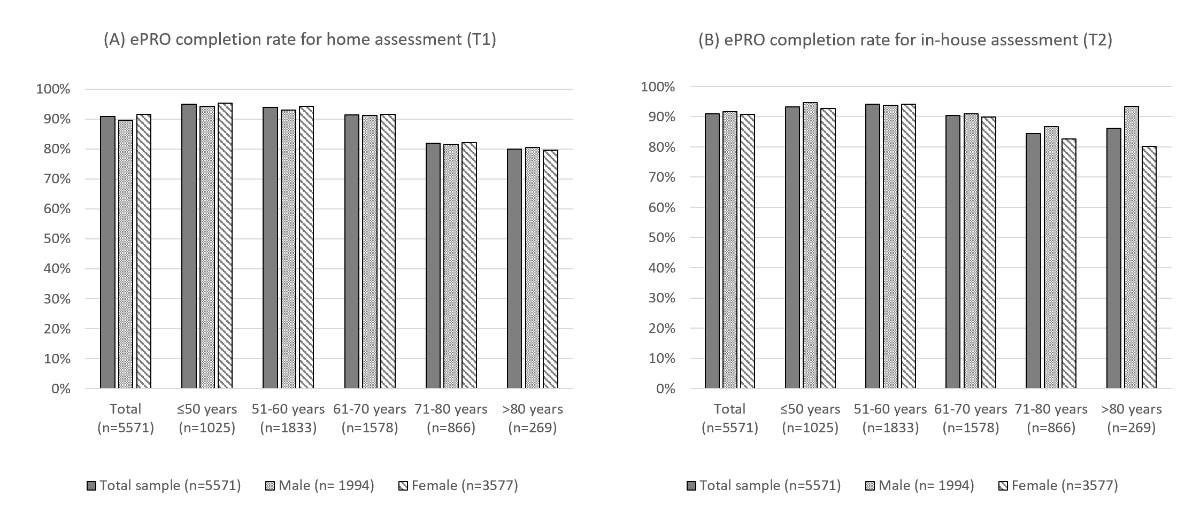
ePRO completion rate across age groups, stratified for gender for (A) home assessment and (B) in-house assessment. ePRO: electronic patient-reported outcome.

Across all age groups, the vast majority of patients (>90%; n=5060/5571) were able to complete the ePRO assessment from home (T1). However, with increasing age, the ePRO completion rates declined: while up to 70 years the ePRO completion rate remained stable at above 90%, there was a clear decrease for patients aged >70 years. The univariate logistic regression showed that the likelihood of not completing the ePRO assessment at home increased with age (Table 2). While overall, a slightly higher ePRO completion rate was observed for women (3273/3577, 91.5% vs 1787/1994, 89.6%; *χ*^2^_1_=5.5; *P*=.02), effect sizes indicate that the effect was of a negligible size (ϕ=0.03). Additionally, no statistically significant gender effect was observed either in patients ≤50 years, (women: 711/746, 95.3% vs men: 263/279, 94.3%; *P*=.49; ϕ=0.02) or in patients between 51 and 60 years (1213/1286, 94.3% vs 508/547, 92.9%; *P*=.24; ϕ=0.03), 61-70 years (825/902, 91.5% vs 616/676, 91.6%; *P*=.81; ϕ=0.01), 71-80 years (408/497, 82.1% vs 301/369, 81.6%; *P*=.84; ϕ=0.01), or older than 80 years (116/147, 79.5% vs 99/123, 80.5%; *P*=.83; ϕ=0.01). For details see Figure 1A.

**Table 2:** Univariable and multivariable logistic regression analysis for the ability for ePRO^a^ home assessment (0=ePRO completion; 1=no ePRO completion).

Variable		Univariable models			Multivariable model	
		OR^b^ (95% CI)	*P* value	OR (95% CI)		*P* value
**Age (years) (reference:** ≤**50)**						
	51-60	0.81 (0.57-1.13)	.21	0.89 (0.63-1.26)		.51
	61-70	0.55 (0.40-0.77)	<.001	0.69 (0.49-0.98)		.04
	71-80	0.24 (0.17-0.33)	<.001	0.32 (0.23-0.45)		<.001
	>80	0.21 (0.14-0.31)	<.001	0.30 (0.19-0.46)		<.001
**EORTC QLQ-C30 scales**						
	Physical functioning^c^	1.30 (1.22-1.39)	<.001	1.22 (1.14-1.30)		<.001
	Role functioning^c^	0.95 (0.90-0.99)	.02	0.97 (0.93-1.02)		.19
	Social functioning^c^	0.91 (0.88-0.95)	<.001	0.93 (0.89-0.97)		<.001
	Fatigue^d^	1.14 (1.07-1.20)	<.001	1.13 (1.06-1.20)		<.001
	Nausea or vomiting^d^	0.94 (0.89-1.00)	.03	0.93 (0.87-0.98)		.007
	Dyspnea^d^	0.94 (0.91-0.98)	.001	0.95 (0.92-0.98)		.003
	Appetite loss^d^	0.95 (0.92-0.99)	.01	0.97 (0.94-1.01)		.16
**Cancer type (reference: Mamma [C50]; International Classification of Diseases, Tenth Revision)**						
	Hemoblastoses (C81-85, C90-96)	0.72 (0.52-1.01)	.056	0.87 (0.61-0.87)		.42
	Prostate (C61)	1.17 (0.77-1.78)	.47	1.36 (0.88-1.36)		.17
	Uterus or ovary (C53-56)	0.56 (0.39-0.82)	.002	0.74 (0.50-0.74)		.14
	Colon (C18-19)	0.65 (0.44-0.97)	.03	0.75 (0.49-0.75)		.18
	Head and neck (C00-14; C30-C32)	0.63 (0.42-0.94)	.03	1.10 (0.71-1.10)		.68
	Lung (C33-C34)	0.97 (0.56-1.69)	.92	1.08 (0.61-1.08)		.78
	Rectum (C20-21)	0.53 (0.32-0.89)	.02	0.83 (0.49-0.83)		.51
	Stomach (C16)	0.99 (0.71-1.39)	.97	1.11 (0.78-1.11)		.56

^a^ePRO: electronic patient-reported outcomes.

^b^OR: odds ratios. ORs for the EORTC QLQ-C30 scores represent 10-point changes.

^c^Higher scores for EORTC QLQ-C30 functioning scales indicate better functioning.

^d^Higher levels for symptom scales indicate a higher symptom load.

A similar picture was found for the in-house assessment at the end of treatment: up to 70 years, over 90% (4125/4436) of patients were able to complete the electronic PRO assessment. The rate decreased to 84.4% (731/866) in patients between 71 and 80 years and 86.2% (232/269) in patients older than 80 years. There was no general gender difference in the ePRO in-house completion rate observable (1827/1994, 91.6% vs 3243/3577, 90.7%; *P*=.23; ϕ=0.02). While in the group of patients >80 years or older men showed a higher ePRO in-house completion rate than women with a small effect size (115/123, 93.5% vs 117/146, 80.1%; *P*=.002; ϕ=0.19), this gender effect could not be observed in patients ≤50 years (264/279, 94.6% vs 692/746, 92.8%; *P*=.29; ϕ=0.03), 51-60 years (513/547, 93.8% vs 1212/1286, 94.2%; *P*=.70; ϕ<0.01), 61-70 years (615/676, 91% vs 811/902, 89.9%; *P*=.48; ϕ=0.02), or 71-80 years (320/369, 86.7% vs 411/497, 82.7%; *P*=.11; ϕ=0.06). For details see Figure 1B.

### Need for Assistance With Electronic Completion

At T1, older patients had a higher need for assistance with the ePRO home assessment prior to admission compared to younger patients: While patients younger than 50 years only needed assistance with the ePRO use in 6.4% (64/993) of cases, this percentage almost doubled in patients 51-60 years (197/1746, 11.3%), while almost two-thirds (159/253, 62.8%) of patients >80 years needed assistance with the ePRO system. While there was no overall gender difference in the need for assistance (371/1895, 19.6% vs 685/3422, 20%; *P*=.70; ϕ<0.01), women older than 70 years reported significantly more often to having needed assistance to complete the ePRO assessment both in the age groups of patients 71-80 years (215/482, 44.6% vs 96/350, 27.4%]; *χ*^2^_1_=25.6; *P*<.001; ϕ=0.18) and >80 years (102/141, 72.3% vs 57/112, 50.9%; *χ*^2^_1_=12.3; *P*<.001; ϕ=0.22). If patients had received help with the assessment at home, they most frequently reported to have received help from their children (519/1124, 46.2%), partners (235/1124, 20.9%), other relatives (152/1124, 13.5%), or others (eg, friends and others, 218/1124, 19.4%).

**Figure 2:**
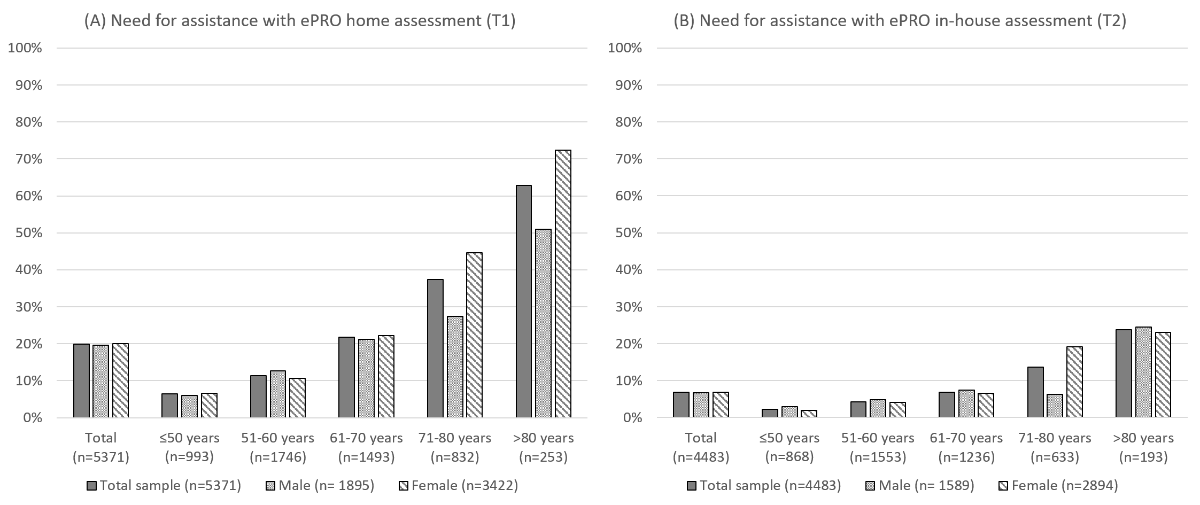
Rate of patients who needed assistance to complete the ePRO assessment at home (before rehabilitation) and at the rehabilitation center (end of rehabilitation) across age groups, stratified for gender. ePRO: electronic patient-reported outcome.

A similar pattern could be observed for the ePRO assessment at the rehabilitation center at the end of treatment: while overall, no significant gender difference could be observed (107/1589, 6.7% vs 197/2948, 6.8%; *P*=.93; ϕ<0.01), women between 71 and 80 years significantly more often needed assistance than men in that age group (70/364, 19.2% vs 17/269, 6.3%; *χ*^2^_1_=21.8; *P*<.001; ϕ=0.19). However, this difference was not observed for patients >80 years (*P*=.93). For details see Figure 2. Most patients who needed help with the ePRO assessments in-house received help from health care personnel (275/304, 90.5%), partners (12/304, 3.9%), children (11/304, 3.6%), friends (5/304, 1.6%), or other relatives (1/304, 0.3%).

**Figure 3:**
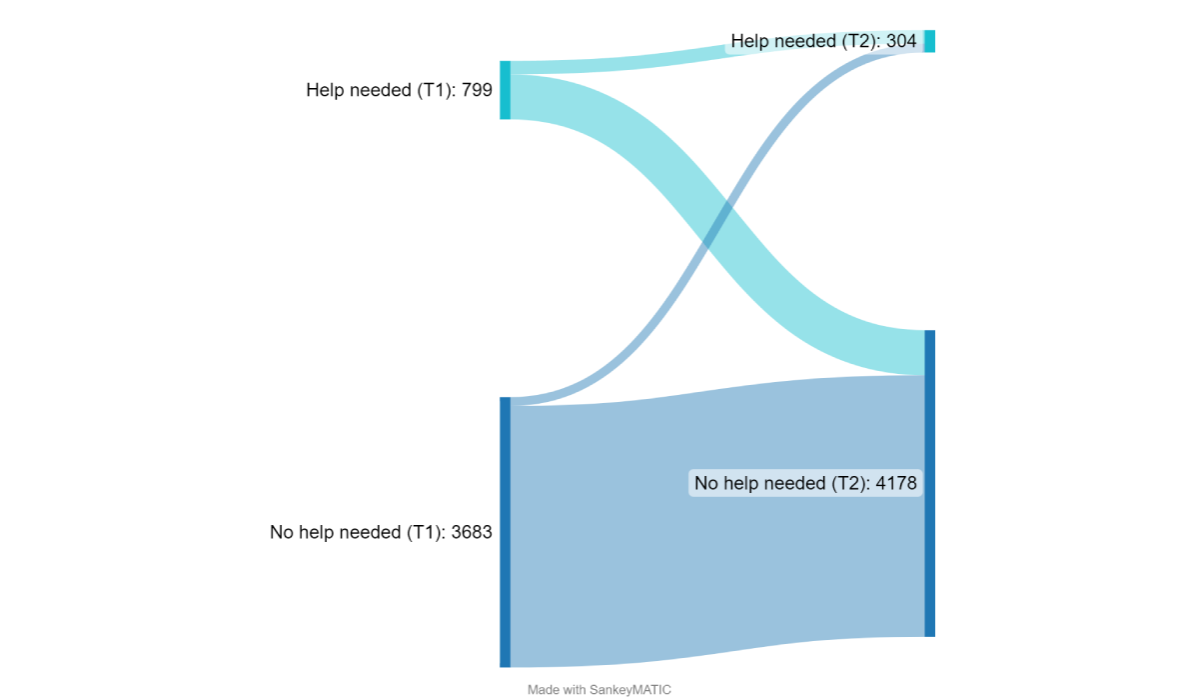
Sankey flow diagram to display the number of patients who needed assistance to complete the ePRO assessment at home before the inpatient rehabilitation (T1) and at the end of the rehabilitation in the rehabilitation center (T2).

The need for assistance did not change for the majority of patients before and after treatment (3747/4482, 83.6%). As described in the methods, data on the need for assistance with the ePRO assessments from both time points were available for patients admitted after January 2018 (n=4482), and only patients from whom the need for assistance was assessed in both T1 and T2 were included in this part of the analyses. At T1, 799 patients needed help with the ePRO assessment at home, while at T2 304 patients needed assistance with the in-house assessment. However, given the different administration modes, a direct comparison of the 2 time points is not reasonable. For details on the need for assistance of patients with complete data sets regarding the need for assistance at both time points see Figure 3 (n=4482).

**Figure 4:**
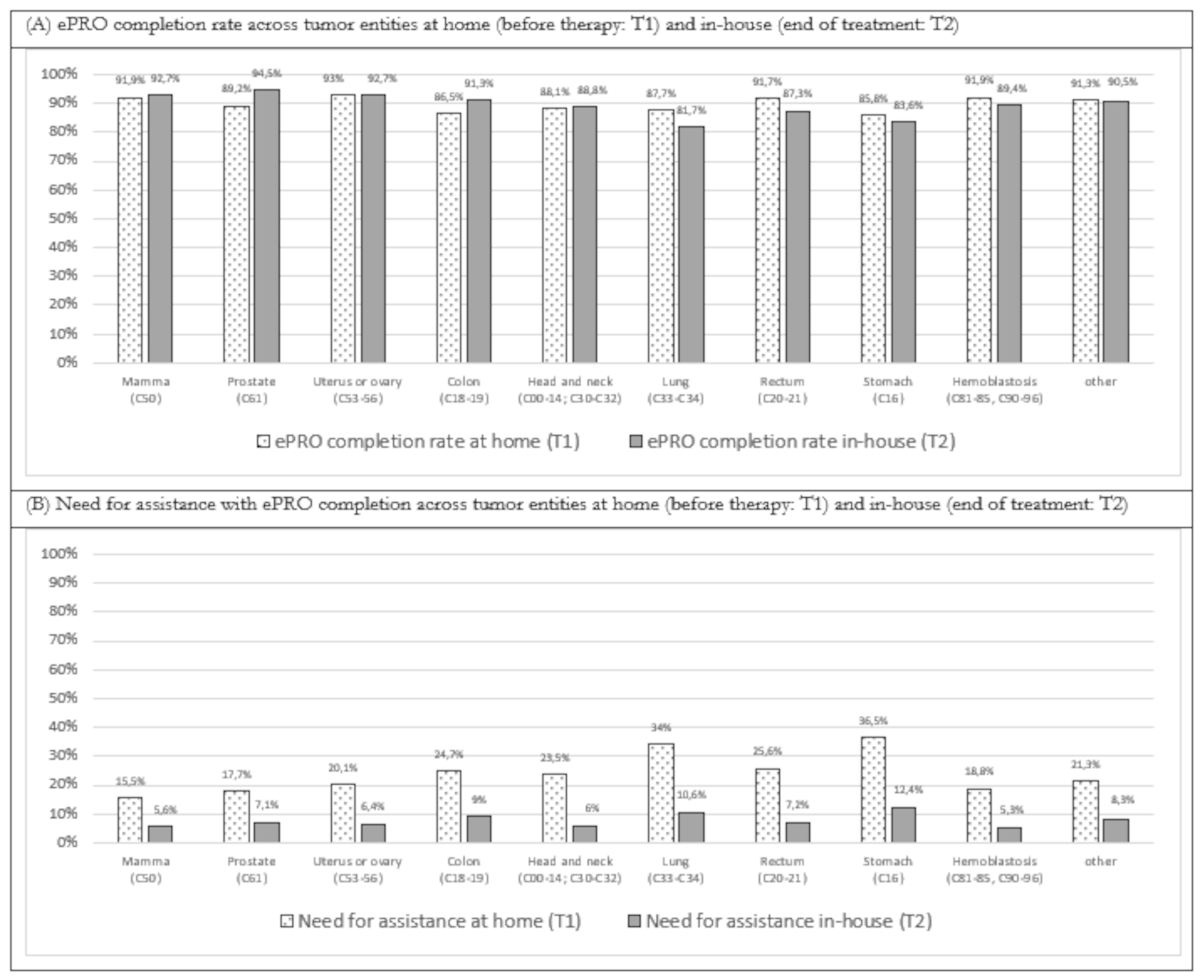
ePRO assessment rate stratified for ICD-10 diagnoses for (A) home assessment (T1) and (B) in-house assessment (T2). ePRO: electronic patient-reported outcome.

Regarding differences across tumor types, the highest ePRO assessment rate at home (T1) was observed in patients with uterine or ovarian cancer (93%), breast cancer (91.9%), and hemoblastosis (91.9%). On the other hand, the lowest completion rates were found in patients with stomach cancer (85.8%) and colon cancer (86.5%). Although the disparities in ePRO completion rates across tumor entities were statistically significant at T1 (*χ*^2^_8_=23.9; *P*=.004; ϕ=0.07), the effect size calculations suggested that the difference was negligible. At the end of treatment, the highest completion rates were observed in patients with prostate cancer (94.5%), uterine or ovarian cancer (92.7%), and breast cancer (92.7%). The differences in ePRO completion rates across tumor entities at the end of treatment (T2) were statistically significant with a small effect size (*χ*^2^_8_=58.2; *P*<.001; ϕ=0.10). For further details, refer to Figure 4A.

Regarding the need for assistance, patients with stomach cancer (46/126, 36.5%) and lung cancer (81/238, 34%) required assistance with the ePRO assessment at home most frequently, whereas patients with breast cancer (307/1977, 15.5%) and hemoblastosis (103/548, 18.8%) needed assistance the least frequently. The difference between cancer entities was statistically significant with a small effect size (*χ*^2^_8_=88.1; *P*<.001; ϕ=0.13). A similar pattern was observed for the ePRO assessment at the end of treatment, where patients with stomach cancer (12/97, 18.8%) and lung cancer (19/179, 10.6%) also required the most support. While the difference between cancer entities remained statistically significant at T2, the effect size calculations suggested that the difference was negligible (*χ*^2^_8_=19.4; *P*=.02; ϕ=0.07). For further details, refer to Figure 4B.

### Time Needed to Complete ePRO Questionnaires

On average, patients needed 4.99 (SD 3.20) minutes to complete the EORTC QLQ-C30 at the first assessment and 4.00 (SD 2.32) minutes at the second assessment, which was significantly faster (t_4480_=107.857; *P*<.001). More than two-thirds of patients (3098/4482, 69.1%) needed less time in the second assessment compared to the first assessment and only 30.9% (1385/4482) needed more time in the second assessment. There were no differences by sex in the completion time for the first assessment (men vs women; 4.9 vs 4.9 min; t_4480_=–0.297; *P*=.77), but a significant difference was found in the second assessment, where men took longer to complete the questionnaires compared to women (4.2 vs 3.9 min; t_4480_=5044; *P*<.001). Notably, at the first assessment, 95.1% (4263/4482) of all patients were able to complete the EORTC QLQ-C30 in under 10 minutes.

The time needed to complete the EORTC QLQ-C30 increased with age between the different age groups with 4.44 (SD 3.09) minutes for the ≤50 years group and up to 6.23 (SD 2.66) minutes in the >80 years age group at T1 (see Table S1 in Multimedia Appendix 1 for the complete data). All between-group differences in completion time were statistically significant at both time points (all *P*<.02) indicating a linear trend.

### Predictors of the ePRO Completion Rate and the Need for Assistance

To investigate whether sociodemographic or clinical variables predicted the ePRO completion rate, logistic regression analyses were conducted. In the first step, univariate analyses were conducted separately for age, the QLQ-C30 subscales, and ICD-10 diagnoses. Significant variables were entered into the final multivariate analyses.

The final model to identify associations with the ePRO completion rate was statistically significant (*χ*^2^_20_=230.3; *P*<.001) and explained 8.8% of the variance. Increasing age was associated with a lower ePRO completion rate: patients >80 years were 3.3 times more likely not to complete the ePRO home assessment compared to patients aged ≤50 years. Additionally, higher physical functioning, lower nausea, and dyspnea as well as higher fatigue and lower social functioning scores were associated with a higher likelihood of completing the ePRO assessment at home. For details, see Table 2.

As for the need for assistance, the final model was statistically significant (*χ*^2^_21_=1013.9; *P*<.001) and explained 17.4% of the variance. Increasing age was clearly associated with a higher need for assistance: patients 80 years or older had a 17.6 times increased likelihood of needing assistance compared to patients younger than 50 years. Lower physical functioning, emotional functioning, and global quality of life as well as higher role and social functioning were associated with a higher likelihood of needing assistance with the ePRO assessment. Additionally, patients with lung cancer and rectum cancer were more likely to need assistance with the ePRO assessment than patients with breast cancer. For details, see Table 3. See Multimedia Appendix 2 for the detailed results of the multivariable logistic regression analyses for the ability for ePRO home assessment and the need for assistance.

**Table 3:** Univariable and multivariable logistic regression analysis for the need for assistance during the ePRO^a^ home assessment. Need for assistance: 0=no; 1=yes.

Variable		Univariate models			Multivariable model	
		OR^b^ (95% CI)	*P* value	OR (95% CI)		*P* value
**Age (years) (reference:** ≤**50)**						
	51-60	1.85 (1.38-2.48)	<.001	1.72 (1.27-2.34)		<.001
	61-70	4.04 (3.05-5.35)	<.001	3.67 (2.73-4.94)		<.001
	71-80	8.67 (6.49-11.58)	<.001	7.21 (5.27-9.85)		<.001
	>80	24.55 (17.14-35.17)	<.001	17.56 (11.92-25.88)		<.001
**EORTC QLQ-C30 scales**						
	Physical functioning^c^	0.65 (0.62-0.69)	<.001	0.73 (0.69-0.77)		<.001
	Role functioning^c^	1.13 (1.09-1.17)	<.001	1.10 (1.06-1.14)		<.001
	Social functioning^c^	1.07 (1.04-1.11)	<.001	1.07 (1.03-1.10)		<.001
	Emotional functioning^c^	0.96 (0.93-0.99)	.04	0.92 (0.88-0.96)		<.001
	Global quality of life^c^	0.90 (0.86-0.95)	<.001	0.93 (0.88-0.99)		.02
	Pain^d^	1.05 (1.01-1.08)	.01	1.04 (1.01-1.08)		.01
	Appetite loss^d^	1.04 (1.01-1.06)	.006	1.01 (0.98-1.04)		.47
	Financial difficulties^d^	0.97 (0.94-0.99)	.006	1.03 (1.00-1.06)		.03
**Cancer type (reference: Mamma [C50]; International Classification of Diseases, ICD-10)**						
	Hemoblastoses (C81-85, C90-96)	1.17 (0.89-1.53)	.26	0.90 (0.67-1.22)		.50
	Prostate (C61)	1.37 (1.03-1.81)	.03	1.01 (0.74-1.39)		.94
	Uterus or ovary (C53-56)	1.79 (1.32-2.41)	<.001	1.09 (0.77-1.55)		.61
	Colon (C18-19)	1.67 (1.23-2.28)	.001	1.23 (0.87-1.74)		.25
	Head and neck (C00-14; C30-C32)	2.81 (2.09-3.77)	<.001	1.31 (0.93-1.83)		.12
	Lung (C33-C34)	1.87 (1.30-2.69)	.001	1.68 (1.12-2.54)		.01
	Rectum (C20-21)	3.13 (2.13-4.59)	<.001	1.68 (1.08-2.61)		.02
	Stomach (C16)	1.26 (0.98-1.61)	.07	1.05 (0.80-1.39)		.72

^a^ePRO: electronic patient-reported outcomes.

^b^OR: odds ratios. ORs for the EORTC QLQ-C30 scores represent 10-point changes.

^c^Higher scores for EORTC QLQ-C30 functioning scales indicate better functioning.

^d^Higher levels for symptom scales indicate a higher symptom load.

## Discussion

### Principal Findings

The aim of this study was to investigate whether adult patients from different age cohorts were able to complete routine ePRO assessments in a large sample of survivors of cancer. Additionally, we analyzed the number of patients across different age groups who required assistance to complete the ePRO assessment and identified factors associated with independent ePRO completion.

In our sample, the majority of patients (5060/5571, >90%) were able to complete the ePRO assessment at home before rehabilitation treatment. However, the ability to complete the ePRO assessment at home decreased with age. Patients up to 70 years old had a completion rate above 90%, but there was a clear decrease in patients older than 70 years. Nevertheless, approximately 80% (924/1135) of geriatric patients were capable of completing the ePRO assessment. In other words, 4 out of 5 geriatric patients were able to complete the assessment. These results are encouraging given the numerous studies that have highlighted the benefits of using ePROs in oncology [44-46].

Although the usefulness of digital tools in oncology is widely recognized, adopting modern technology devices in geriatric patient samples presents several challenges. First, older patients are often hesitant to embrace new technologies due to perceived complexity [47]. However, the number of older patients using modern technology devices has steadily increased and is expected to continue to do so with the growing familiarity of older adults with technology and the expanding population. A pan-European study on the use of modern technology by senior citizens revealed that as early as 2015, 40%-50% of participants 65 years or older used the internet almost daily [48]. According to the Pew Research Center [49], in 2021, approximately 61% of adults aged 65 years and older owned smartphones compared to just 18% in 2013 [50], and an Austrian 2020 panel showed that 49.8% of adults aged 65 years or older used smartphones [51].

Second, some technologies, such as touchscreens, may pose a barrier for older patients [52]. However, touchscreen devices can also be easier to use for patients with visual impairments since they allow for the adaptation of font size and background coloring, which is not possible with traditional paper-pencil questionnaires. Despite the known challenges with touchscreen interfaces compared to computers using a keyboard and mouse, the performance gap between older and younger adults is significantly less pronounced with touchscreen devices [53]. This indicates that touchscreen devices may even be an attractive option for older patients. Additionally, answering questions on a tablet reduces the likelihood of patients accidentally skipping a question (or even entire pages) in a paper-pencil questionnaire because items can be displayed as single items on the screen.

We also investigated the time it took patients to complete a standardized questionnaire electronically as a surrogate marker of the feasibility of the assessments and ease of use. Almost all patients (4263/4482, 95.1%) were able to complete the questionnaire, the QLQ-C30 electronically in below 10 minutes without having received prior dedicated training (aside from the instruction leaflet they received via postal services). Considering that the average completion time of the paper-pencil version of the QLQ-C30 is around 11 minutes [54], this highlights that ePRO assessments can be more time-efficient and potentially easier to use if support is supplied to those patients who need it. The average completion time of 4.9 (SD 3.20) minutes is similar to other studies that measured the time of electronic completion for the QLQ-C30 [55,56]. Another finding was that patients needed less time to complete the questionnaire at the second assessment, indicating a potential learning effect. However, we cannot rule out that this effect might be linked to the change in modality, that is, remote versus in-house assessments.

In our sample, we found that approximately 37% (311/832) of patients 71-80 years and 63% (159/253) >80 years needed assistance with completing the ePRO assessment at home. For the home ePRO completion rate, most patients received support from their direct relatives (ie, children or partners). We observed a clear gender difference in those age groups, with women reporting a substantially higher need for assistance than men. This is consistent with epidemiological research from Austria, which reported a higher use of the internet among older men than women (61% vs 47%) [57]. A potential explanation for this gender difference may be found in the higher percentage of long-standing professional activity of men compared to women in the oldest age cohort. However, a longitudinal observational study from Switzerland showed that the gender effect may become less pronounced over time: while in 2014, older women used the internet significantly less often than men, no gender difference was observed in 2019 [58].

The substantially lower need for assistance in the rehabilitation center compared to the initial ePRO assessment could be caused by different factors. Either this can be explained by patients becoming more accustomed to the assessment—in terms of fluid internet literacy—or the limiting factor may be found in the steps required to register and log into the patient portal for the first time, rather than the ePRO completion itself. In the rehabilitation center, these steps were not necessary, since patients were handed a tablet that already displayed the correct site.

Apart from the clear and stable association of lower age with higher home assessment rates and less need for help, we also observed an association between the patients’ self-reported functioning and symptom levels. A 10-point difference in physical functioning was associated with a 20% higher likelihood of completing the ePRO assessment at home and a 37% lower likelihood of needing assistance to complete the ePRO assessment. This is in line with epidemiological studies, which frequently associated better functioning and quality of life levels with higher internet literacy and use of modern technology [59,60]. Interestingly, other factors, such as cognitive functioning, were not associated with either the ePRO home assessment rate or the need for help to complete the home assessment. Both the ePRO completion rate and the need for assistance were found to be marginally associated with the underlying cancer type. Patients with lung cancer and stomach cancer exhibited the lowest ePRO completion rates and the highest need for help during the assessment. This phenomenon could be attributed to the greater symptom burden associated with the diseases and treatments experienced by these patient groups, who reported the lowest levels of physical functioning and the highest levels of fatigue, nausea, and pain compared to all other cancer types [10,61].

While, in general, research on completion rates and associated factors is still scarce for patients with cancer, there are some relevant related studies in other patient populations. However, there is mixed evidence on which factors or patient groups are associated with noncompliance to responding to surveys. For example, 1 study found that older patients were less likely to respond to surveys (among patients treated surgically for prostate disease [62], while other studies report no differences in the completion of follow-up surveys based on age or sex (eg, in orthopedic ePRO follow-up assessments [63]). Finally, another study with a diverse hospital population found that either very young or very old patients had a higher chance of being nonresponding [64]. Such mixed results indicate that factors for noncompletion likely differ between patient populations and the respective ePRO system and use cases. This highlights the need for more research to better understand different patients’ motivations (or lack thereof) to participate in ePRO assessments.

### Implications for Improving ePRO Assessment Completion Rates

Our findings provide useful information on the factors (age, sex, and self-reported functioning or symptoms) that are associated with reduced ePRO assessment completion and the need for help. This information is valuable for future research and the use of ePROs in clinical practice as an example of what completion rates can be expected from patients of different sex and age groups. Moreover, it is important to consider how the factors that influence home assessment rates can be addressed in the clinical setting to improve care. Further, 1 approach is to provide or encourage adequate support pathways. In our study, older patients received help from relatives to complete the assessments prior to visiting the rehabilitation center. If patients are made aware that completing an ePRO assessment at home is considered an integral part of their treatment, this can increase the chance that patients who require support actively look for it. Setting up an adequate support hotline for patients who require help is another potential way to help patients complete ePRO assessments.

For patients who are already at the hospital or, in our case, rehabilitation, finding ways to increase their self-efficacy with eHealth care (like ePRO follow-up assessments) may be a key factor for future participation. Research shows that, among others, a lack of self-efficacy is an important barrier to using eHealth applications in general [65]. If patients are encouraged to use electronic systems like CHES during their stay and successfully do so (or receive support if required), this may increase their motivation to also participate in future remote assessments.

### Limitations

Due to the retrospective character of this study, several important factors could not be taken into account. For one, no data on the technology- and health-literacy of the sample were available. While we consider the home ePRO completion rate as a relatively good indicator for these concepts, we cannot draw causal conclusions from the presented data. Additionally, we had no information on the socioeconomic status, level of education, potential language barriers, or migration background of the included sample, all of which have been identified as potential predictors for usage of modern technologies [57]. While we had information on the proportion of patients that needed help with the assessment, no information is available as to why the patients needed help (ie, logging in, completion of questionnaires, and adaption of font size). Since the patients received a uniform treatment, it was not possible to determine whether specific treatments contributed to the improvement of the ePRO assessment rate. More data from qualitative studies are needed to gain insight in the above-mentioned points.

### Conclusions

To our knowledge, this is the first study to investigate the ability of older survivors of cancer to complete ePRO assessments at home as well as at an inpatient facility. Our results clearly indicate that despite the remaining skepticism regarding the ability of older individuals to participate in ePRO assessments, a large proportion of the older patients were indeed capable of self-reporting their health using modern technology devices. However, when ePRO assessments are planned for older patients, it seems that patients require help (eg, from relatives) to conduct home-based assessments, or that it is more feasible to conduct assessments in-house.

## Appendix

Multimedia Appendix 1 Completion times in minutes for the electronic completion of the EORTC QLQ-C30 at both assessment time points by age group.

Multimedia Appendix 2 Detailed results of multivariable logistic regression analysis for the ability for ePRO home assessment and the need for assistance.

## Abbreviations

CHES: Computer-based Health Evaluation System
EORTC QLQ: Organization for the Research and Treatment of Cancer Quality of Life Questionnaire
ePRO: electronic patient-reported outcome
HRQOL: health-related quality of life
PRO: patient-reported outcome

## Data Availability

The data sets analyzed in this paper are not publicly available, because of ethical and legal restrictions (data contain potentially identifying and sensitive patient information). If not already reported within this work, the authors may provide descriptive data on individual medical indicators for admission and discharge or the expected change due to in-patient health care for various groups and diagnoses. Requests for access to anonymized data sets should be directed to the corresponding author.
