# Supplementary material for: Usability of Electronic Patient-Reported Outcome Measures for Older Patients With Cancer: Secondary Analysis of Data from an Observational Single Center Study
Source: J Med Internet Res. 2023 Sep 21;25:e49476. doi: 10.2196/49476 (PMC10557001; doi:10.2196/49476)
Supplement: Multimedia Appendix 1 [file jmir_v25i1e49476_app1.docx]

**Table S1.** Completion times in minutes for the electronic completion of the EORTC QLQ-C30 at both assessment time points by age group.

|  | T1 | | T2 | | average time improvement (T1-T2) * | |
| --- | --- | --- | --- | --- | --- | --- |
|  | n=5555 | | n=5045 | | n=4907 | |
| Age group | mean | SD | mean | SD | mean improvement | SD |
| ≤50 years | 4.44 | 3.09 | 3.55 | 3.21 | 0.86 | 3.33 |
| 51-60 years | 4.82 | 3.06 | 3.80 | 2.11 | 0.97 | 3.12 |
| 61-70 years | 5.08 | 3.45 | 3.99 | 1.74 | 1.01 | 3.31 |
| 71-80 years | 5.47 | 3.17 | 4.56 | 1.94 | 0.77 | 3.05 |
| >80 years | 6.23 | 2.66 | 5.67 | 2.72 | 0.46 | 3.02 |
| total | 4.99 | 3.20 | 4.00 | 3.54 | 0.91 | 3.20 |

**Note.** Time is given in minutes. SD = standard deviation; * only patients where the completion time for T1 and T2 were available were included in the time improvement analysis.
