# Supplementary material for: Usability of Electronic Patient-Reported Outcome Measures for Older Patients With Cancer: Secondary Analysis of Data from an Observational Single Center Study
Source: J Med Internet Res. 2023 Sep 21;25:e49476. doi: 10.2196/49476 (PMC10557001; doi:10.2196/49476)
Supplement: Multimedia Appendix 2 [file jmir_v25i1e49476_app2.docx]

**Table S1.** Multivariable logistic regression analysis for the ability for ePRO home assessment.

|  | Regression coefficient B | Standard error | Wald | df | Sig. | Odds Ratio (OR) | 95% Confidence Intervall for OR | |
| --- | --- | --- | --- | --- | --- | --- | --- | --- |
|  |  |  |  |  |  |  | Lower Value | Upper Value |
| Age (reference: ≤50 years) |  |  | 66.751 | 4 | <.001 |  |  |  |
| 51-60 years | -0.126 | .196 | 0.412 | 1 | .52 | .882 | .600 | 1.296 |
| 61-70 years | -0.499 | .195 | 6.583 | 1 | .01 | .607 | .414 | .889 |
| 71-80 years | -1.125 | .203 | 30.767 | 1 | <.001 | .325 | .218 | .483 |
| >80 years | -1.422 | .255 | 31.004 | 1 | <.001 | .241 | .146 | .398 |
| EORTC QLQ-C30 scales |  |  |  |  |  |  |  |  |
| Physical functioning ^a^ | 0.197 | .041 | 23.310 | 1 | <.001 | 1.217 | 1.124 | 1.319 |
| Role functioning ^a^ | -0.032 | .028 | 1.276 | 1 | .26 | .969 | .917 | 1.023 |
| Social functioning ^a^ | -0.068 | .025 | 7.711 | 1 | .005 | .934 | .890 | .980 |
| Emotional functioning ^a^ | 0.128 | .035 | 13.568 | 1 | <.001 | 1.137 | 1.062 | 1.217 |
| Global quality of life ^a^ | -0.127 | .034 | 13.712 | 1 | <.001 | .881 | .824 | .942 |
| Pain ^b^ | -0.046 | .021 | 4.930 | 1 | .03 | .955 | .916 | .995 |
| Appetite loss ^b^ | -0.004 | .024 | 0.035 | 1 | .85 | .996 | .951 | 1.043 |
| Cancer Type (ref: Mamma (C50) |  |  | 12.909 | 9 | .17 |  |  |  |
| Hemoblastoses (C81-85, C90-96) | -0.067 | .204 | 0.109 | 1 | .74 | .935 | .627 | 1.394 |
| Prostate (C61) | 0.324 | .256 | 1.610 | 1 | .21 | 1.383 | .838 | 2.282 |
| Uterus / Ovary CA (C53-56) | -0.222 | .238 | 0.876 | 1 | .35 | .801 | .502 | 1.276 |
| Colon (C18-19) | -0.562 | .235 | 5.731 | 1 | .02 | .570 | .360 | .903 |
| Head and neck (C00-14; C30-C32) | 0.216 | .287 | 0.566 | 1 | .45 | 1.241 | .707 | 2.178 |
| Lung (C33-C34) | -0.287 | .299 | 0.925 | 1 | .34 | .750 | .418 | 1.347 |
| Rektum CA (C20-21) | -0.335 | .315 | 1.135 | 1 | .29 | .715 | .386 | 1.325 |
| Stomach (C16) | -0.023 | .205 | 0.013 | 1 | .91 | .977 | .654 | 1.461 |
| Cancer Type (ref: Mamma (C50) | 0.100 | .172 | 0.337 | 1 | .56 | 1.105 | .788 | 1.550 |
| Constant | 0.972 | .420 | 5.363 | 1 | .02 | 2.644 |  |  |
| *ePRO = electronic patient reported outcomes (0 = ePRO completion; 1= no ePRO completion); ref = reference; ^a^ higher scores for EORTC QLQ-C30 functioning scales indicate better functioning; b higher levels for symptom scales indicate a higher symptom load; ORs for the EORTC QLQ-C30 scores represent 10-point changes* | | | | | | | | |

**Table S2.** Multivariable logistic regression analysis for need for assistance during the ePRO home assessment.

|  | Regression coefficient B | Standard error | Wald | df | Sig. | Odds Ratio (OR) | 95% Confidence Intervall for OR | |
| --- | --- | --- | --- | --- | --- | --- | --- | --- |
|  |  |  |  |  |  |  | Lower Value | Upper Value |
| Age (reference: < 50 years) |  |  | 293.599 | 4 | <.001 |  |  |  |
| 50-60 years | 0.600 | .173 | 11.958 | 1 | .001 | 1.821 | 1.297 | 2.559 |
| 60-70 years | 1.345 | .172 | 61.408 | 1 | <.001 | 3.838 | 2.742 | 5.373 |
| 70-80 years | 2.130 | .182 | 137.643 | 1 | <.001 | 8.415 | 5.895 | 12.011 |
| >80 years | 3.031 | .227 | 178.021 | 1 | <.001 | 20.722 | 13.276 | 32.346 |
| EORTC QLQ-C30 scales |  |  |  |  |  |  |  |  |
| Physical functioning | -0.325 | .031 | 111.326 | 1 | <.001 | 0.723 | 0.680 | 0.767 |
| Role functioning | 0.079 | .022 | 13.163 | 1 | <.001 | 1.082 | 1.037 | 1.129 |
| Social functioning | 0.061 | .020 | 9.070 | 1 | .003 | 1.063 | 1.022 | 1.107 |
| Emotional functioning | -0.083 | .024 | 11.720 | 1 | .001 | 0.920 | 0.878 | 0.965 |
| Global quality of life | -0.040 | .033 | 1.541 | 1 | .21 | 0.960 | 0.901 | 1.024 |
| Pain | 0.047 | .020 | 5.814 | 1 | .02 | 1.049 | 1.009 | 1.090 |
| Appetite loss | 0.012 | .016 | 0.575 | 1 | .44 | 1.012 | 0.981 | 1.045 |
| Cancer Type (ref: Mamma (C50) |  |  | 10.184 | 9 | .34 |  |  |  |
| Hemoblastoses (C81-85, C90-96) | -0.099 | .171 | 0.334 | 1 | .56 | 0.906 | 0.649 | 1.266 |
| Prostate (C61) | -0.124 | .188 | 0.433 | 1 | .51 | 0.884 | 0.611 | 1.278 |
| Uterus / Ovary CA (C53-56) | 0.062 | .204 | 0.093 | 1 | .76 | 1.064 | 0.713 | 1.588 |
| Colon (C18-19) | 0.218 | .203 | 1.155 | 1 | .28 | 1.243 | 0.836 | 1.850 |
| Head and neck (C00-14; C30-C32) | 0.426 | .201 | 4.480 | 1 | .03 | 1.531 | 1.032 | 2.272 |
| Lung (C33-C34) | 0.403 | .244 | 2.727 | 1 | .10 | 1.496 | 0.928 | 2.412 |
| Rektum CA (C20-21) | 0.233 | .270 | 0.745 | 1 | .39 | 1.262 | 0.744 | 2.140 |
| Stomach (C16) | 0.001 | .166 | 0.000 | 1 | .99 | 1.001 | 0.723 | 1.385 |
| Cancer Type (ref: Mamma (C50) | 0.071 | .133 | 0.284 | 1 | .59 | 1.074 | 0.827 | 1.394 |
| Constant | 0.733 | .297 | 6.095 | 1 | .01 | 2.081 |  |  |
| *ePRO = electronic patient reported outcomes (0 = ePRO completion; 1= no ePRO completion); ref = reference; ^a^ higher scores for EORTC QLQ-C30 functioning scales indicate better functioning; b higher levels for symptom scales indicate a higher symptom load; ORs for the EORTC QLQ-C30 scores represent 10-point changes* | | | | | | | | |
